# Reassessing the curvature effect in tables and chairs

**DOI:** 10.1177/20416695251341682

**Published:** 2025-05-22

**Authors:** Erick G. Chuquichambi, Tobiasz Trawinski, Enric Munar, Letizia Palumbo

**Affiliations:** 1 Human Evolution and Cognition Group (EvoCog), 16745 University of the Balearic Islands, Palma, Balearic Islands, Spain; 2 School of Psychology, 4588 Liverpool Hope University, Liverpool, UK

**Keywords:** object category, curvature, visual preference, eye movements

## Abstract

Several studies have consistently demonstrated that people generally prefer curved over angular contours. However, the magnitude of the curvature effect varies across stimuli, for example, with a larger effect reported for abstract stimuli compared to interior spaces. A comparison across stimuli that share similar physical features and belong to the same categories is warranted to determine whether curvature is a basis of object preference. Another important question is whether inspection differences, based on contour and object category, affect object preference. In Experiment 1, we addressed these questions by recording eye movements as participants rated their preferences for images of two types of common-use objects: tables and chairs. In Experiment 2, we limited the stimuli presentation to 84 ms, as brief presentations are thought to enhance the curvature effect. Neither of the two experiments confirmed a clear preference for curvature in tables or chairs. Yet, curvature significantly influenced fixation durations, with curvilinear tables eliciting longer fixations than rectilinear ones, although without affecting overall preference. The findings are discussed in the context of familiarity and object functionality in shaping preference judgements.

## How to cite this article

Chuquichambi, E.G., Trawinski, T., Munar , E., & Palumbo , L. (2025). Reassessing the curvature effect in tables and chairs . *i-Perception*, *16*(0), 1-20. https://doi.org/10.1177/20416695251341682

## Introduction

Preference for smooth curvature has been the subject of investigation since the late 19th century (Cotter et al., 2017; Gómez-Puerto et al., 2016). From the curvilinear contours of abstract patterns (Bertamini et al., 2016, 2019) to the smooth curvature of manufactured objects (Bar & Neta, 2006, 2007; Chuquichambi et al., 2021), artworks (Munar et al., 2023), architectural spaces (Ruta et al., 2019; Vartanian et al., 2019), and curvature elicits positive responses. Accordingly, numerous studies have consistently demonstrated that people exhibit a robust preference for curved stimuli over angular ones, attributing qualities such as beauty, harmony, and positive emotional resonance to items characterized by smooth contours (Palumbo & Bertamini, 2016; Palumbo et al., 2021).

Since smooth curvature represents a special case of good continuation, collinearity, and good Gestalt principles (Kanizsa, 1979), the preference for curved stimuli is usually attributed to perceptual fluency (Reber et al., 2004). Specifically, it is argued that curved stimuli possess inherent perceptual advantages, facilitating enhanced processing fluency irrespective of the stimulus context (Field et al., 1993). Indeed, when examining the processing of curved and angular shapes matched for their complexity, curved shapes are processed faster than their angular counterparts across a range of tasks including categorization, same-different judgement, rotation, symmetry detection, and speeded response (Bertamini et al., 2016, 2019; Chuquichambi et al., 2020; Palumbo et al., 2015). These phenomena support a domain-general mechanism where the preference for smooth curvature could result from the human visual system tuned to curvature (Yue et al., 2020).

Nevertheless, despite extensive empirical evidence supporting the generalizability of the curvature effect, some contrary results show that this preference varies as a function of specific conditions. For example, Corradi et al. (2019) reported that the strongest curvature effect on preference was found for abstract shapes presented briefly. In addition, while a high preference for curvature in short-presentation durations was confirmed in a recent meta-analysis of 61 studies by Chuquichambi et al. (2022), the analysis also revealed that this preference is moderated by stimulus category, expertise, and task.

One potential explanation for these discrepancies in research findings is that when dissimilar objects from different categories are confronted, information beyond just the contour is available for the viewer to use in forming a preference judgement. This could explain why the effect size of curvature is most pronounced for abstract shapes and decreases with more diverse objects such as interior space designs (Chuquichambi et al., 2022; Palumbo et al., 2022; Tawil et al., 2021). Therefore, to determine whether curvature is a primary factor influencing preference, it is essential to compare objects that belong to similar categories but are more ecologically valid than abstract shapes.

To address this issue, we selected two sets of common-use objects, tables and chairs, that share similarities in both object category (furniture) and physical features (both typically have four legs and at least one plane). The primary manipulation was the contour of the objects, curvilinear versus rectilinear. If the contour type determines liking, we would expect a preference for curvilinear objects over rectilinear ones, regardless of the category. In contrast, if an interaction between contour and category is observed, despite the similarity of the objects, it would suggest that curvature alone does not determine preference. This finding would be in line with previous research indicating that the effect of contour type may be more pronounced in specific categories (Gao & Soranzo, 2020; Soranzo et al., 2018).

The second question of interest is whether the way we visually inspect objects varies depending on their contour and category and whether such differences could explain the observed liking responses. For example, Leek et al. (2012) demonstrated that observers tend to fixate on collinear elements along a contour path, suggesting that contour integration actively guides eye movements. Additionally, the number and duration of fixations increase as the contour becomes more angular, indicating that greater effort is required to integrate the most salient points along angular contours (Van Humbeeck et al., 2013). This increased cognitive demand implies that angular shapes require longer processing times and therefore anchor attention more effectively. These results suggest that recording and analysing the number and duration of fixations may provide insights into how curvature influences visual inspection.

On the other hand, non-eye-tracking research suggests that curvy shapes are processed faster than angular ones across various tasks (Bertamini et al., 2016, 2019; Chuquichambi et al., 2020). The less efficient processing of angular shapes may result from attention being drawn to regions near corners, which carry the most critical information about the shape of an object (Bertamini et al., 2013; Cole et al., 2007). In terms of eye movements, this could mean longer fixation durations on areas with sharp angles. Supporting this idea, evidence from word–object pair preference tasks shows similar patterns. For example, Cheung et al. (2024) found that the first fixation duration on words was longer for angular, less-preferred fonts compared to more curved, preferred fonts.

We conducted two experiments to investigate these two questions. In Experiment 1, participants were asked to make their preference judgement on a set of images (tables and chairs) while their eye movements were recorded. Complementary, in Experiment 2, we explored the relationship between preference for curvature and visual inspection by manipulating presentation time. Previous studies have shown that the curvature effect is stronger with brief stimulus presentations (Corradi et al., 2019). Therefore, stimuli were presented either for a brief period (84 ms) allowing approximately one fixation, or in long (until-response) display time conditions. We predict that the preference for curvature will be more pronounced in the short display time condition.

In sum, the main objective of this study was to assess the extent to which object contour (curvilinear vs. rectilinear) explains liking for commonly used objects comparable in terms of object category. The second question of interest was to examine how viewers visually inspect objects while forming the liking judgements. Specifically, we investigated whether visual inspection, indexed by the number of fixations and mean fixation duration, differs based on object contour and category and whether these differences could account for the preference of commonly used objects. Finally, we explored whether the stimulus presentation influences curvature preference in our stimulus set. Both experiments presented here were pre-registered on the OpenScienceFramework (OSF; https://doi.org/10.17605/OSF.IO/QT2GA).

## Experiment 1

### Method

#### Participants

Forty students (32 female, *M*_age_ = 21.45, SD_age_ = 4.96) from Liverpool Hope University participated in course credits. All participants reported normal or corrected to normal vision. The study was conducted following the code of practice of the British Psychological Society (BPS) guidelines and received ethical approval (Protocol number: PSYJM7939) from the Committee for Ethics in Research of the [BLINDED]. All participants gave written informed consent before the experiment.

The final sample size was determined based on a priori power analysis for linear mixed models using the simr package in R (Green & MacLeod, 2016; Judd et al., 2017). First, we simulated a dataset considering a 2 (Category: chair vs. table) × 2 (Curvature: rectilinear vs. curvilinear) within-subject design. The dataset included a sample of 10 participants providing 80 trials for a total of 800 observations. Second, we employed the population parameters with a grand mean equal to 50 which correspondents to the average liking ratio, a small positive coefficient for the category variable (*β* = .20), and a large coefficient for the contour variable (*β* = .90). The selection of these coefficients followed the results from a meta-analysis of preference for curvature literature (Chuquichambi et al., 2022). We then applied mixed-effects modelling to the simulated dataset. Liking ratings were included as the outcome variable, and the variables’ contour, category, and their interaction were specified as fixed effects. Participants and stimuli were included as random effects and random intercepts were specified for each random effect. Next, based on the model’s results, we ran the simulation using the powerSim function with 1,000 simulations and the alpha error level was set to 0.05. Last, we estimated a power curve with 1,000 simulations and Satterthwaite approximation to determine statistical significance. The desired test power of 80% was achieved with 40 participants (*M* = 84%, 95% CI [75.32, 90.57]).

#### Apparatus

The experiment was designed using the SR Research Experiment Builder (SR Research Ltd., Mississauga, Ontario, Canada). The task was presented on a BenQ XL2420 T monitor, with a screen size of 53 cm×30 cm. Participants were seated at a distance of 98 cm giving a visual angle of 30.26° by 17.40° for the screen. The screen resolution was 1920 × 1080 with a refresh rate of 100 Hz. Viewing was binocular, but only movements of the right eye were recorded using an SR Research Limited Eye-Link 1000 eye tracker operating at 1000 Hz. Head movement was stabilized using a chin and headrest. Participants responded by using the computer mouse.

#### Stimuli

**Figure 1.**
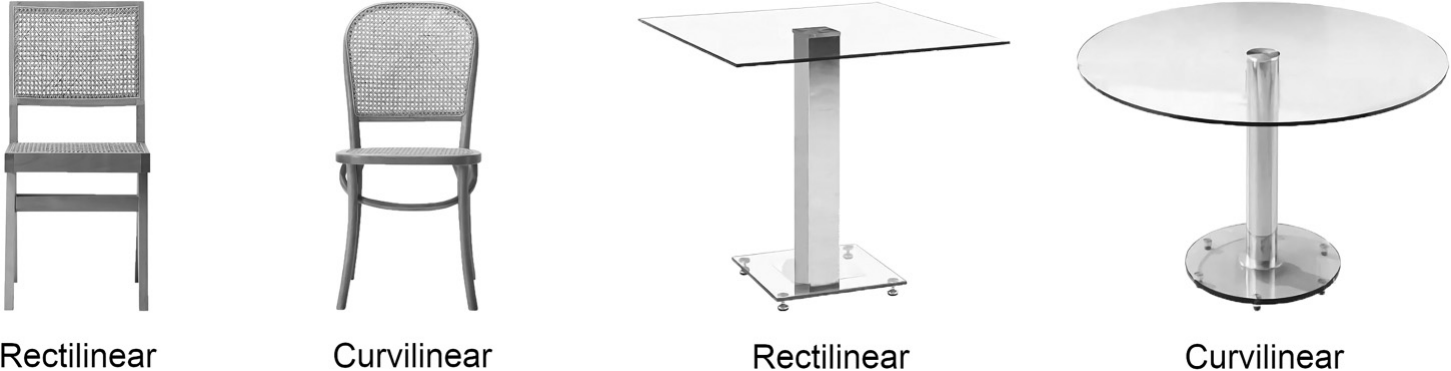
Examples of the pairs of chairs and tables images used in the experiment.

A total of 80 greyscale images were used in this experiment. The high-resolution images were uploaded from the Google Image Search. The stimuli set consisted of 40 images of chairs and 40 images of tables. These objects were selected because of their similarities in both object category and physical features (both typically have four legs and at least one plane). Moreover, among other kinds of furniture, tables and chairs are two of the most effective elements in defining interior space dimensions (Von Castell et al., 2014). The image set mainly varied on the curvature dimension meaning that half of the chair and table images had a curvilinear design, and the other half had a rectilinear design. They have also been organized in pairs to match their curvilinear and rectilinear versions in style, materials, and intended use (see Figure 1). Each stimulus was carefully edited using Adobe Photoshop CS6 to remove any aspect that could differentiate the two versions of each pair except for their contour type. The complete set of stimuli is available on OSF: https://osf.io/v69fx/.

**Figure 2.**
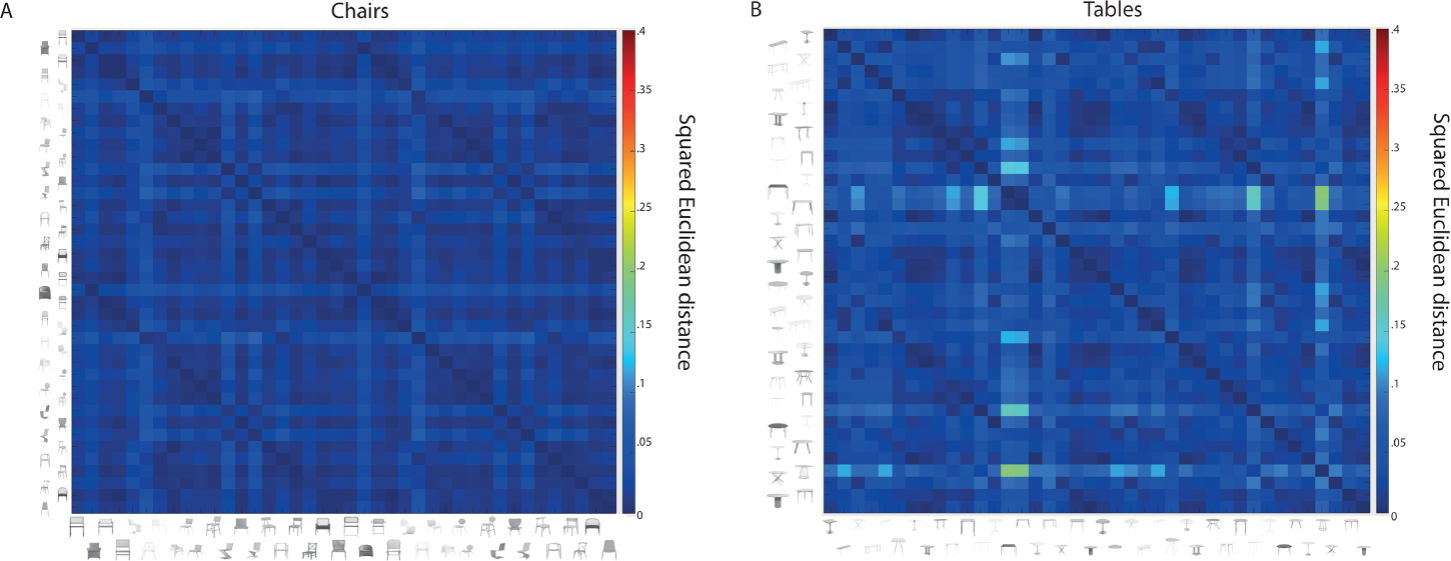
Pairwise dissimilarity (squared Euclidean distance) of gist statistics of chairs (A) and tables (B) images. Darker blue indicates lower dissimilarity between the stimuli, while lighter colours indicate higher dissimilarity.

To exclude any possible differences in the basic visual properties of the images, a gist descriptor was calculated for each image. The gist descriptor refers to a set of perceptual dimensions representing the spatial structure of scenes, including visual low-level properties (He et al., 2020; Oliva & Torralba, 2001, 2006; Rice et al., 2014). Specifically, a series of Gabor filters across eight orientations and four spatial frequencies was applied to the images. Each of the images was segmented into a 4 × 4 grid and the energy was averaged within each grid to obtain final gist statistics containing 80 values. Lastly, pairwise dissimilarities of the gist statistics were calculated across images within and between object category and contour, by squared Euclidean distance (Figure 2). As expected, results revealed no significant differences among the curvilinear and rectilinear versions of tables and chairs images (*t*(778) = −0.54, *p* = 0.59, 95% CI [−0.0024, 0.0013]; *t*(778) = 0.15, *p* = 0.88, 95% CI [−0.0036, 0.0042], respectively).

Next, root-mean-square contrast (the standard deviation of the pixel intensities) was calculated as an index of the objective complexity of the images (Marin & Leder, 2013). This measure was considered because previous studies have shown significant relationships between measures of objective complexity and subjective ratings, such as pleasantness or beauty (Forsythe et al., 2011; Marin & Leder, 2013). The results confirmed no significant differences in objective complexity neither between the rectilinear (*M* = 43.09; SD = 23.19) and curvilinear (*M* = 40.47; SD = 19.94) chairs nor between the rectilinear (*M* = 47.42; SD = 18.88) and curvilinear (*M* = 46.45; SD = 18.20) tables (*t*(38) = 0.17, *p* = 0.87, 95% CI [28.6, 65.2] and *t*(38) = 0.37, *p* = 0.71, 95% CI [20.4, 63.1], respectively).

Finally, we controlled for the average market prices of each object, as price variations could reflect differences in materials, quality, and perceived value (Shi et al., 2021). An independent *t*-test confirmed no significant differences among the actual average market prices of the chairs with a rectilinear (*M* = £422, SD = 712) and a curvilinear design (*M* = £317, SD = 417; *W* = 201, *p* = 0.99, 95% CI [−199, 939]). Similarly, there were no significant differences among the average market price of the tables with a rectilinear (*M* = £1237; SD = 1409) and a curvilinear design (*M* = £1570, SD = 1686; *t*(36) = −0.66, *p* = 0.51, 95% CI [−139, 2942]).

To quantify the perceptual attributes of curvature, novelty, complexity, weight, and interest of selected images, a different group of 35 participants (27 female, *M*_age_ = 22.83 years, SD_age_ = 10.74) was presented with 80 images in random order via an online experiment designed with Qualtrics (Provo, UT). As described in the pre-registration, the final sample size for this experiment was determined based on previous studies that examined the effect of multiple measures such as interest, complexity, or familiarity, among others on preference judgement (Carbon, 2010; Ruta et al., 2019). Participants’ task was to rate the design of each object in terms of curvature (from 1 = rectilinear to 100 = curvilinear), novelty (from 1 = familiar to 100 = novel), complexity (from 1 = simple to 100 = complex), and light (from 1 = heavy to 100 = light). We also asked participants to rate each object in terms of Interestingness (from 1 = boring to 100 = interesting) as interest is often associated with an event’s novelty–complexity (e.g., evaluating an object as novel, unexpected, complex, curious, or surprising; Berlyne, 1949, 1960) and comprehensibility (i.e., understanding of the event based on one’s skills, knowledge, and resources). Interestingness captures additional perceptual attributes, as participants may assess an object as interesting due to its novelty and comprehensibility, despite it not necessarily being perceived as pleasant (Silvia, 2005, 2008). Each image was presented on a white background of 500 pixels × 500 pixels. In each trial, participants were shown an image one at a time until response.

The results of 2 × 2 analysis of variances (category: chairs vs. tables by contour: curvilinear vs. rectilinear) revealed that chairs were rated as more novel, complex, and lighter than tables (*F* = 6.97, *p* = 0.010, *η_p_*^2^ = 0.084; *F* = 9.25, *p* = 0.003, *η_p_*^2^ = 0.11; *F* = 39.25, *p* < 0.001, *η_p_*^2^ = 0.34, respectively). Critically, there was no significant difference between chairs and tables in terms of perceived curvature and interestingness (*F* = 0.40, *p* = 0.53; *F* = 2.72, *p* = 0.10, respectively). The results also confirmed that the curvilinear objects were perceived as more curvilinear and lighter than the rectilinear objects (*F* = 479.45, *p* < 0.001, *η_p_*^2^ = 0.86; *F* = 4.06, *p* = 0.048, *η_p_*^2^ = 0.051, respectively). No significant differences were found between the curvilinear and rectilinear objects in terms of novelty, complexity, and interestingness (*F* = 0.04, *p* = 0.85; *F* = 0.05, *p* = 0.83; *F* = 0.86, *p* = 0.36, respectively). The only significant interaction was found for perceived curvature (*F* = 10.46, *p* = 0.002, *η_p_*^2^ = 0.12), indicating that the difference between curvilinear and rectilinear objects was slightly larger in tables (mean deviation [MD] = 45.05, *t* = 17.77, *p* < 0.001) than chairs (MD = 33.46, *t* = 13.20, *p* < 0.001). Descriptive statistics are shown in Table 1.

**Table 1.** Descriptive statistics for the attributes obtained in the online experiment.

Measure	Contour			
	Chairs		Tables	
	Curvilinear mean (SD)	Rectilinear mean (SD)	Curvilinear mean (SD)	Rectilinear mean (SD)
Curvature	62.98 (10)	29.52 (5.94)	69.91 (8.72)	24.86 (6.78)
Novelty	47.39 (15.79)	45.48 (18.77)	37.64 (10.11)	38.30 (10.82)
Complexity	46.32 (13.15)	45.69 (19.97)	36.48 (11.21)	35.67 (12.49)
Light	59.11 (7.11)	52.86 (8.07)	43.78 (11.51)	41.42 (10.81)
Interestingness	48.31 (13.15)	45.53 (15.76)	43.48 (10.66)	40.99 (10.59)

#### Procedure

The validated set of stimuli was used in the main eye tracker part of Experiment 1. The stimuli were presented centrally on the computer screen against a white background. The height of the objects was equalized to 13.8 cm giving a visual angle of 8.11°. Widths varied between 6.5 and 25.3 cm giving visual angles between 3.81° and 14.54°.

Participants were asked to provide their liking of the stimuli while their eye movements were recorded. The experiment began with a 9-point calibration procedure for accurate eye movement recording. The eye tracker was calibrated to <.5° error. Trials started with a fixation point centred on the screen. Once this point was fixated, an image of a chair or table was presented centrally on the screen. After 2000 ms a horizontal sliding bar appeared under the image and remained on the screen until a response was made. Participants were asked to judge their liking of the furniture on a scale (from 1 = dislike to 100 = like) by moving the slider on the sliding bar. The intertrial interval was 500 ms. Participants rated all 80 images and the order in which the images were presented was randomized. Prior to the main task, participants completed six practice trials corresponding to six images from a different furniture category (i.e., wardrobes).

### Results

#### Data Analysis

Analyses were conducted in R Version 4.4.1 (R Core Team, 2024). Data were fitted in linear mixed-effect models (LMMs) using the lme4-package (Bates et al., 2015), and MASS-package (Venables & Ripley, 2002). These models simultaneously consider both the between-subjects and within-subjects effects of the independent variables (Baayen et al., 2008). The inferential statistics were obtained using the afex package (Singmann et al., 2016). Predicted marginal means, contrasts, and confidence intervals were calculated as estimates of each fixed effect and its associated uncertainty using the lsmeans package (Lenth, 2016; Version 4.1). The random effects were structured for items and participants including slopes for meaningful fixed effects and correlation. The full random structure was trimmed down for those models that did not converge or had a high or equal to zero correlation (Barr et al., 2013; Brauer & Curtin, 2018). The *t*-values equal to 1.96 or higher were interpreted as significant because the *t*-statistic in LMMs approximates the *z*-statistic for high degrees of freedom (Baayen et al., 2008).

The results are structured to address to what extent preference judgements, as well as visual processing, might be modulated by the object features and object categories of commonly used objects. We did so by examining the effect of the object category (chair vs. table), contour (rectilinear vs. curvilinear), and perceived curvature on liking ratings, number of fixations, and mean fixation duration. These measures were 10×log(*x*) to increase the normality of the data distribution (Kliegl et al., 2009). Two models were computed for each measure: contour and perceived curvature. In the first set of models, the fixed factors were category and contour. In the second set of models, the fixed factors were category and perceived curvature. The interactions between these variables were also included in the models. In addition, the models estimated the magnitude of these effects while controlling for the object’s novelty, complexity, lightness, and interestingness. That is, these variables were continuous measures and included in the models as covariates. All continuous variables were mean-centred before analyses. The random structure for the analyses was (1 + category | subject) + (1 | stimuli) for liking rating and number of fixations. The intercept-only model was conducted for analyses of mean fixation duration. The results of the first set of analyses are presented in Table 2 and the second in Table 3.

**Table 2.** Estimates for the effects and interactions between category, contour, and covariates from the mixed-effect model (LMM) for liking ratings, number of fixations, and mean fixation duration.

	Experiment 1								
	Liking rating			Number of fixations			Mean fixation duration		
Measure	*β*	*SE*	*t*	β	*SE*	*t*	β	*SE*	*t*
Intercept	35.45	0.60	**59**.**19**	18.73	0.44	**42**.**44**	52.67	0.44	**118**.**58**
Category	−0.90	0.51	−1.77	0.17	0.12	1.47	0.08	0.08	**1**.**07**
Contour	0.46	0.31	1.48	0.001	0.07	0.01	−0.07	0.06	−1.22
Category × Contour	0.43	0.29	1.47	−0.05	0.06	−0.75	0.13	0.06	**2**.**26**
Novelty	−0.11	0.05	**−2**.**11**	0.005	0.01	0.43	−0.006	0.01	−0.56
Complexity	−0.12	0.06	**−2**.**05**	−0.02	0.01	−1.25	0.01	0.01	1.01
Lightness	−0.06	0.03	−1.82	−0.01	0.01	−1.79	0.006	0.006	0.98
Interestingness	0.27	0.07	**3**.**84**	0.01	0.02	0.86	−0.007	0.013	−0.54

*Note*. Significant effects are indicated in bold.

**Table 3.** Estimates for the effects and interactions between category, perceived curvature, and covariates from the mixed-effect model (LMM) for liking ratings, number of fixations, and mean fixation duration.

	Experiment 1								
	Liking rating			Number of fixations			Mean fixation duration		
Measure	*β*	*SE*	*t*	*β*	*SE*	*t*	*β*	*SE*	*t*
Intercept	35.44	0.60	**58**.**79**	18.73	0.44	**42**.**45**	52.66	0.44	**118**.**62**
Category	−0.84	0.52	−1.62	0.17	0.11	1.49	0.09	0.08	1.20
Perceived curvature	−0.007	0.017	−0.45	−0.0002	0.003	−0.05	0.005	0.003	1.59
Category × Perceived curvature	−0.005	0.015	−0.30	0.004	0.003	1.36	−0.006	0.003	**2**.**27**
Novelty	−0.11	0.054	**−2**.**04**	0.003	0.01	0.28	−0.003	0.01	−0.27
Complexity	−0.11	0.06	−1.80	−0.014	0.01	−1.14	0.010	0.011	0.95
Lightness	−0.07	0.03	**−2**.**06**	−0.013	0.007	−1.81	0.005	0.006	0.81
Interestingness	0.25	0.07	**3**.**40**	0.013	0.02	0.83	−0.010	0.014	−0.76

*Note*. Significant effects are indicated in bold.

#### Liking Rating

The results did not reveal a significant main effect of Contour on liking ratings. The design of the rectilinear (*M* = 35.9, 95% CI [34.6, 37.3]) and curvilinear objects (*M* = 35, 95% CI [33.6, 36.3]) did not differ significantly. Similarly, the main effects of Category at its interaction with Contour were non-significant. Regarding the additional attributes, increasing interestingness predicted higher liking ratings, while increasing novelty and complexity (i.e., decreasing familiarity and simplicity) predicted lower liking ratings.

In the second model, the Perceived Curvature did not influence liking ratings. Similarly, the interaction between Perceived Curvature and Category was non-significant. Additionally, interestingness and novelty again significantly predicted liking and an increase in lightness was associated with lower liking ratings.

#### Data Analysis

Analyses were conducted in R Version 4.4.1 (R Core Team, 2024). Data were fitted in linear mixed-effect models (LMMs) using the lme4-package (Bates et al., 2015), and MASS-package (Venables & Ripley, 2002). These models simultaneously consider both the between-subjects and within-subjects effects of the independent variables (Baayen et al., 2008). The inferential statistics were obtained using the afex package (Singmann et al., 2016). Predicted marginal means, contrasts, and confidence intervals were calculated as estimates of each fixed effect and its associated uncertainty using the lsmeans package (Lenth, 2016; Version 4.1). The random effects were structured for items and participants including slopes for meaningful fixed effects and correlation. The full random structure was trimmed down for those models that did not converge or had a high or equal to zero correlation (Barr et al., 2013; Brauer & Curtin, 2018). The *t*-values equal to 1.96 or higher were interpreted as significant because the *t*-statistic in LMMs approximates the *z*-statistic for high degrees of freedom (Baayen et al., 2008).

The results are structured to address to what extent preference judgements, as well as visual processing, might be modulated by the object features and object categories of commonly used objects. We did so by examining the effect of the object category (chair vs. table), contour (rectilinear vs. curvilinear), and perceived curvature on liking ratings, number of fixations, and mean fixation duration. These measures were 10 × log(*x*) to increase the normality of the data distribution (Kliegl et al., 2009). Two models were computed for each measure: contour and perceived curvature. In the first set of models, the fixed factors were category and contour. In the second set of models, the fixed factors were category and perceived curvature. The interactions between these variables were also included in the models. In addition, the models estimated the magnitude of these effects while controlling for the object’s novelty, complexity, lightness, and interestingness. That is, these variables were continuous measures and included in the models as covariates. All continuous variables were mean-centred before analyses. The random structure for the analyses was (1 + category|subject) + (1|stimuli) for liking rating and number of fixations. The intercept-only model was conducted for analyses of mean fixation duration. The results of the first set of analyses are presented in Table 2 and the second in Table 3.

#### Number of Fixations

There was no significant evidence of an influence of contour, perceived curvature, and category on the number of fixations.

#### Mean Fixation Duration

**Figure 3.**
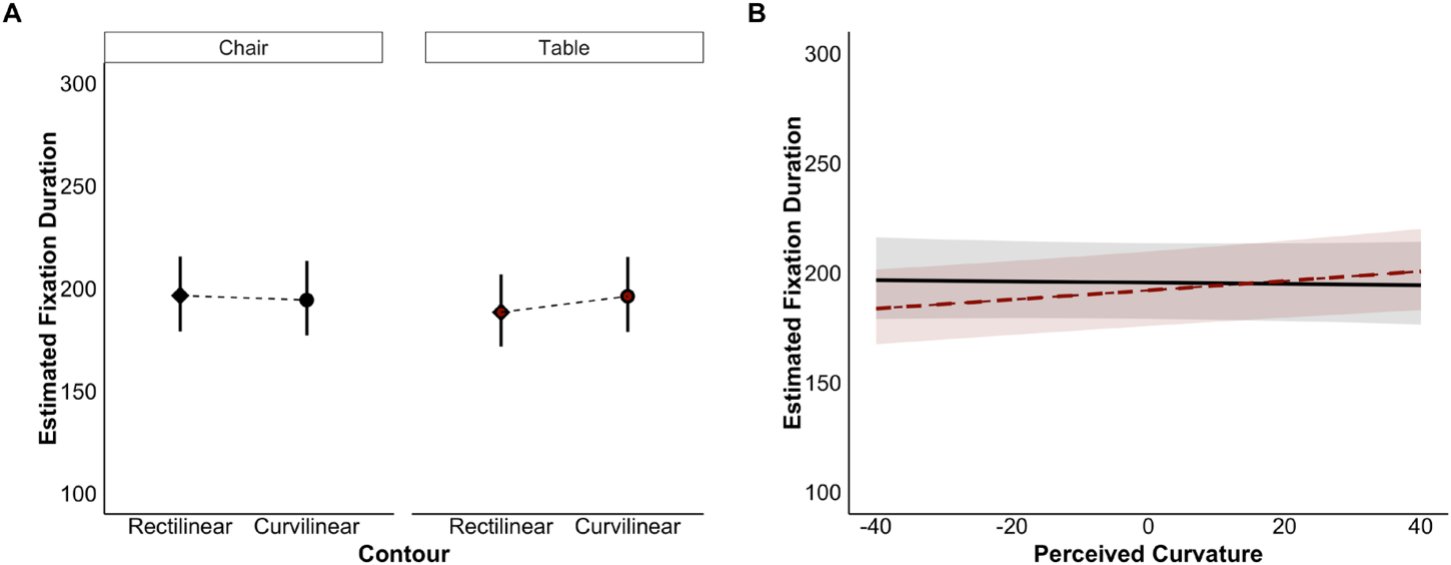
Interaction effects between contour and category (A) and perceived curvature and category (B) on the average estimated mean fixation duration. Panel (A) shows the estimated fixation duration for the chairs (black) and tables (dark red) with rectilinear and curvilinear designs. Panel (B) shows the predicted fixation durations based on the perceived curvature for chairs (black line) and tables (dark red dashed line). The figure shows untransformed values for fixation duration. Error bars (A) and ribbons (B) represent 95% CIs.

The results showed that the main effects of category and contour on mean fixation duration were not significant. However, the interaction between these variables was significant. The mean fixation duration for the curvilinear tables (*M* = 196 ms, 95% CI [179, 216]) was significantly longer than for the rectilinear tables (*M* = 188 ms, 95% CI [172, 207], *t* = 2.47, 95% CI [0.078, 0.732]). In contrast, the difference in fixation duration between the curvilinear (*M* = 194 ms, 95% CI [177, 213]) and rectilinear (*M* = 196 ms, 95% CI [179, 216]) chairs was non-significant (*t* = −0.66, 95% CI [−0.444, 0.223]) (Figure 3A).

For the model with perceived curvature, only the interaction between perceived curvature and category was significant. Prolonged fixations were associated with increasing perceived curvature for tables, but not for chairs (Figure 3B).

#### Relationship Between Liking and Eye Movements

We now turn to explore the relationship between liking rating, number of fixations, and mean fixation duration. This exploratory analysis addresses the longstanding question of how eye movements relate to preference. We conducted separate LMM analyses with random structure (1 + contour|subject) + (1|stimuli) and mean-centred number of fixations and mean fixation duration. Results indicated that the liking measure was not significantly predicted neither by the number of fixations, *β *= −0.17, *SE* = 0.32, *t* = −0.53, nor by the mean fixation duration, *β *= −0.29, *SE* = 0.46, *t* = −0.64, or interaction between them, *β *= −0.22, *SE* = 0.22, *t* = −0.97.

### Discussion

The results of Experiment 1 revealed that the contour of the objects did not significantly influence the number of fixations and average fixation time. However, the significant interaction between the category and contour of the objects suggests that curved objects are associated with prolonged fixations within specific categories. Similarly, the perceived curvature of the objects positively predicted the mean fixation duration for tables but not for chairs. Therefore, this suggests that the object category shapes how curved objects are visually inspected, rather than the reverse.

On the other hand, the results did not show a significant relationship between preference and visual processing. This result indicates that the number and duration of fixations may not be directly associated with liking responses for everyday objects, such as those examined in the present study.

The results also showed no significant influence of contour and perceived curvature on liking the design of the objects. These findings were not completely unexpected and aligned with those of recent studies (Chuquichambi et al., 2022; Corradi & Munar, 2020). For instance, Palumbo et al. (2022) found no evidence of the curvature effect when using images of interior spaces with non-experts in design, while a group of quasi-experts in industrial design showed a preference for angular over curved interiors. This supports the idea that the extent to which we like objects is influenced by factors other than contour.

In Experiment 1, stimuli were presented on the screen for at least 2 s, remaining visible until the participants provided their ratings. However, previous research using images of real objects has suggested that the preference for curvature is limited to short presentation times (Chuquichambi et al., 2022; Corradi et al., 2019). Therefore, in Experiment 2, we examined whether the absence of curvature preference in Experiment 1 was influenced by the duration of the stimulus presentation.

## Experiment 2

Experiment 2 consisted of an online liking rating task using two presentation time conditions in a within-subjects design. In the first condition, each object was presented briefly (84 ms), while in the second condition, it was presented until participants responded. In both conditions, participants were asked to rate how much they liked the design of each object. We predicted that the curvature ratings would influence participants’ liking ratings in the short presentation time, but not in the until-response condition (Chuquichambi et al., 2022; Corradi et al., 2019). Additionally, we also expected to replicate the effects of the perceptual attributes found in Experiment 1.

### Materials & Methods

#### Participants

Fifty participants (24 women, *M*_age_ = 32.5 years, SD_age_ = 11.63) were recruited via the prolific recruitment platform (Palan & Schitter, 2018). The sample size was based on a power analysis for linear mixed models following the same procedure as in Experiment 1. The study was conducted following the code of practice of the BPS guidelines and received ethical approval from the Committee for Ethics in Research of Liverpool Hope University.

#### Stimuli and Procedure

Stimuli consisted of the same set of images as in Experiment 1. Participants were presented with 80 images via an online experiment designed with Psytoolkit (Stoet, 2017). The experiment presented each object centrally on the computer screen. Participants were asked to rate their liking of the design of the object on a scale (from 1 = dislike to 100 = like) by clicking on a horizontal sliding bar below the object. The experiment consisted of two conditions with 80 trials in each. In one condition, the object was displayed for 84 ms (Bar & Neta, 2006; Corradi et al., 2019; Leder et al., 2011). Next, it was replaced by a scrambled version of the same object displayed for 84 ms to reduce the interference of perceptual memory for preference judgement (Cazzato et al., 2022; Mele et al., 2013). In the second condition, each object was displayed until participants responded. Once they responded, the scrambled version of the same object was displayed for 84 ms as in the other condition. Condition order and trial sequence were randomized. As in Experiment 1, participants completed six practice trials corresponding to six images from a different furniture category (e.g., wardrobes). Finally, participants were debriefed and thanked.

### Results

#### Data Analysis

Data were analysed using LMMs and following the analytical strategy of Experiment 1. Two models were fitted with a liking rating as the dependent variable. The first model analysed the effects of the category (chair vs. table), contour (curvilinear vs. rectilinear), and condition (84 ms vs. until-response) on liking. The model also included the interactions between these variables. The second model analysed the effects of perceived curvature, and its interaction with category, and condition. As in Experiment 1, the models estimated these effects while including the objects’ novelty, complexity, lightness, and interestingness as continuous measures. All continuous variables were mean-centred before analyses. The random structure of the models was (1 + category|subject) + (1|stimuli). The results are presented in Tables 4 and 5.

**Table 4.** Estimates for the effects and interactions between category, contour, and covariates from the mixed-effect model (LMM) for liking rating in Experiment 2.

	Liking rating		
Measure	*β*	*SE*	*t*
Intercept	34.68	0.78	**44**.**56**
Category	−0.24	0.38	−0.64
Contour	−0.08	0.15	−0.52
Condition	0.42	0.08	**−5**.**38**
Category × Contour	0.10	0.14	0.75
Contour × Condition	0.03	0.08	0.39
Category × Condition	0.24	0.08	**3**.**08**
Novelty	−0.09	0.02	**−3**.**54**
Complexity	−0.02	0.03	−0.69
Lightness	−0.05	0.02	**−2**.**95**
Interestingness	0.014	0.03	0.42

*Note*. Significant effects are indicated in bold.

**Table 5.** Estimates for the effects and interactions between category, perceived curvature, and covariates from the mixed-effect model (LMM) for liking rating in Experiment 2.

	Liking rating		
Measure	*β*	*SE*	*t*
Intercept	34.67	0.78	**44**.**56**
Category	−0.24	0.38	−0.62
Curvature	0.005	0.008	0.61
Condition	0.42	0.08	**5**.**37**
Category × Curvature	−0.006	0.007	−0.83
Curvature × Condition	−0.0004	0.004	−0.11
Category × Condition	0.24	0.08	**3**.**07**
Novelty	−0.084	0.025	**−3**.**39**
Complexity	−0.020	0.028	−0.73
Lightness	−0.047	0.016	**−2**.**92**
Interestingness	0.011	0.034	0.33

*Note*. Significant effects are indicated in bold.

#### Liking Rating

The results of the models showed a significant main effect of condition on liking rating. Specifically, participants liked the design of the objects under the 84 ms condition (*M* = 33.5, 95% CI [28.7, 39]) more than in the until-response condition (*M* = 30.7, 95% CI [26.4, 35.8]). However, the main effects of contour and category on liking were non-significant. The interaction between category and condition was revealed to be significant. Liking difference between the 84 ms and until-response conditions was significant with chairs (*t* = −5.98, 95% CI [−1.76, −0.89]), but not with tables (*t* = −1.62, 95% CI [−0.79, 0.075]). In addition, the results of the perceptual attributes showed that increasing novelty and lightness (i.e., decreasing familiarity and heaviness) predicted lower liking ratings. None of the other effects or interactions was significant.

Regarding the model with perceived curvature, the results showed no main effect of this fixed factor on liking rating. Similarly, its interactions with category and condition were non-significant. The effects of condition and its interaction with category remained significant with a significant difference in liking rating for tables but not for chairs. Finally, novelty and lightness still negatively predicted liking ratings.

### Discussion

Contrary to our expectations, the results of Experiment 2 did not show an effect of preference for curvature in the short presentation time condition, neither for the category factor (curvilinear vs. rectilinear) nor for perceived curvature as rated by participants. There was also no effect in the until-response time condition. These findings did not align with those previously reported in the literature. It might be that other aspects of the stimuli could play an important role in liking responses. For example, repeated exposure to instances of the same two object categories, or the dominance of other variables characterizing object design could have influenced the relative importance of object features such as curvature (Gao & Soranzo, 2020; Soranzo et al., 2024).

On the other hand, and consistent with the results of Experiment 1, there was no significant difference in the liking of the design of tables and chairs. Additionally, increased novelty and lightness were associated with lower liking ratings. In contrast, the relationship between interestingness and liking was not significant in this experiment.

## General Discussion

We investigated the extent to which object preference is driven by object contour and moderated by the interaction between contour and object category. Specifically, we focused on contrasting curvilinear and rectilinear objects within two comparable object categories: tables and chairs. Previous studies have shown that curved contours are generally preferred over sharp-angled contours using different experimental paradigms, types of stimuli, and groups of participants (Chuquichambi et al., 2022; Corradi & Munar, 2020; Gómez-Puerto et al., 2016). However, it has been demonstrated that preference for curvature is not a universal effect. Chuquichambi et al. (2022) found that while the preference for visual curvature is reliable, several factors influence the magnitude of its effect. Some studies have shown that this preference can increase, attenuate, and even disappear under certain conditions, such as viewing time and expertise of the participants (Corradi et al., 2019; Palumbo et al., 2022). This highlights the involvement of other prioritized factors in object preference, such as valence over the contour line (Leder et al., 2011). However, while it is established that preference for curvature is influenced by the conditions under which it is assessed, it remains unclear whether this preference persists in response to more subtle variations. Our first objective was to test whether curvature determines the preference for common-use objects, or whether object category, even when comparable, influences the role of curvature in determining preference.

The second objective was to test whether differences in how viewers inspect objects would account for the liking responses. The prediction was that rectilinear objects would generate longer mean fixation durations, indicating more effortful visual processing, which would result in a lower preference for rectilinear objects. In contrast, shorter mean fixation duration to curved contours would be associated with a higher preference for curvilinear objects. Contrary to our expectations, the results of Experiment 1 did not show a higher preference for curvilinear tables or chairs over rectilinear ones. The same finding was replicated in Experiment 2 where the presentation time included 84 ms and until-response conditions.

Experiment 1 also examined whether there is a relationship between how participants inspect object contours and categories and their preference responses. The results showed that curvilinear objects elicited longer mean fixation durations, but this effect was observed only for tables, and not for chairs. Notably, the interaction between contour and category in visual inspection did not account for the preference responses.

The present findings may be interpreted through the lens of the mere exposure effect (Zajonc, 1968), which suggests that increased exposure to stimuli can enhance preference for those stimuli. Although curved tables elicited longer fixations, this does not necessarily translate into a preference. It is possible that participants were simply more engaged with the curvature of tables due to their less typical design, leading to longer fixation durations without a corresponding increase in preference. Conversely, the lack of difference in fixation durations for chairs suggests that participants may have been less sensitive to differences in the amount of curvature within that category, likely due to greater exposure to both rectilinear and curvilinear chair designs. Ultimately, while visual inspection plays an important role, it does not guarantee that it will be directly linked with preference judgements, especially when other factors, such as mere exposure, may also significantly influence preferences.

An alternative explanation for the absence of a curvature effect in our results can be found in Djebbara and Kalantari's (2023) commentary on the meta-analysis by Chuquichambi et al. (2022). These authors proposed that the *affordances* of stimuli could play a central role in the presence or absence of the curvature effect. The concept of *affordance* refers to the possibility of interactions with an object or the physical world (Gibson, 1986). Using the meta-analysis database from Chuquichambi et al. (2022), Djebbara and Kanteri analysed a subset of studies that presented objects with rich affordances, such as those in virtual reality and real contexts. Their results indicated that the preference for curved contours tends to decrease as the affordances provided in the studies increase. Based on this reanalysis, they suggested that curvature preference may be limited to preference when viewing pictures.

In our two experiments, participants were asked to evaluate the design of everyday objects, such as tables and chairs. This could have led to a more realistic interaction with the stimuli, potentially diminishing the curvature effect due to the increased salience of affordance in the rated objects. Other studies that used images of realistic stimuli, such as interior space designs, have also failed to replicate the curvature effect (Palumbo et al., 2022; Tawil et al., 2021). The findings underscore the relevance of examining additional variables that characterize realistic objects, such as their functionality, which may influence the relative importance of curvature features (Soranzo et al., 2024). However, it is worth noting that Bar and Neta (2006, 2007) used images of everyday objects and found the curvature preference effect, as did other studies using the same stimuli (Corradi et al., 2019; Gómez-Puerto et al., 2016; Munar et al., 2015).

Another possible alternative explanation is that the emphasis of the task on furniture design may have dissipated the effect of preference for curvature. Tinio and Leder (2009) demonstrated that repeated exposure to stimuli can alter the influence of object attributes, such as symmetry and complexity, on preference judgements. Their findings showed that massive exposure to stimuli resulted in higher judgements of beauty for structurally opposite stimuli (Biederman & Vessel, 2006), challenging the notion of structural generalization effects. This phenomenon might partially explain our preference results, as the design of rectilinear objects was rated similarly to that of curvilinear objects. Furthermore, angular or rectilinear tables are encountered more frequently than curved ones in everyday life, with curvilinear designs being recognized as more novel and innovative (Dazkir & Read, 2012; Leder & Carbon, 2005, Lovegrove, 2010). Perhaps participants spent more time inspecting curvilinear tables compared to rectilinear ones due to their lower familiarity with the curvilinear design of these objects. Nonetheless, this longer fixation time did not translate into a higher liking for curvilinear tables. Therefore, while curvature can lead to longer fixation durations in specific object categories, it does not necessarily correlate with a higher preference for the curved object. In our study, the longer fixation time on curved tables could be attributed to factors such as familiarity or functionality. Further studies will need to clarify the trade-off between preferred properties and functionality in object design, and how these factors influence visual processing and liking judgements.

Although not directly related to our hypothesis, we observed that increasing interestingness predicted higher liking ratings. This finding aligns with previous research suggesting that interestingness is a crucial factor in preference evaluation (Berlyne, 1949; Berlyne et al., 1968). When objects or images are perceived as more interesting, they tend to evoke positive emotional responses, leading to higher liking ratings (Graf & Landwehr, 2015). Moreover, our results showed that increasing novelty and complexity negatively impacted liking ratings. This supports the view that the effects of novelty and complexity on preference responses depend on the stimuli category (Marin et al., 2016; Mayer & Landwehr, 2018). This finding is consistent with the principles of mere exposure theory, which proposes that people tend to prefer objects they encounter more frequently in their lives (Martindale et al., 1988; Zajonc, 1968). Finally, the finding that an increase in lightness led to a decrease in liking ratings for both chairs and tables may be explained by the association of objects perceived as heavier with desirable qualities in furniture, such as durability, strength, and solidity. Together, these findings could be linked to the idea that the utility and functionality of common-use objects play a more significant role in shaping preferences than the basic effect of curvature, although this idea remains to be tested.

The finding that participants liked the design of objects more under the 84 ms condition than in the until-response condition suggests that preference judgement can be explained in terms of gist processing. In the 84 ms condition, participants had only enough time for about one fixation, allowing them to capture the essence or overall impression of the objects without focusing on specific details. This quick, broad processing typically emphasizes positive attributes, as the viewer's response is based on an immediate, holistic impression rather than a more deliberate, feature-based analysis. In contrast, the until-response condition allowed participants more time to inspect details more closely, which may have led them to notice aspects they found less appealing, potentially decreasing their liking for the object.

In terms of potential limitations, we acknowledge that our stimulus set is limited to only two specific object categories. Future studies should be run to determine whether our findings generalize to other kinds of stimuli. However, in each case, future studies will need to do their best to select commonly used stimuli that share similarities in both object categories and physical features.

Moreover, it might be that the design of the stimuli, which did not allow for testing for objects that are fully curvilinear or rectilinear could limit the generalizability of the findings. That is, some objects included curvilinear and rectilinear features in different parts of their design. However, our estimates of curvature were determined from participant ratings as in other studies (e.g., Vartanian et al., 2024). This seems appropriate given that the primary concern is the viewer's experience of object contour. Nevertheless, future studies should also consider additional formal measures of object contour using curvature estimation algorithms (e.g., Walther et al., 2023). However, standardized operational definitions and computational metrics of curvature remain a topic of debate in the literature (Watier, 2024). Likewise, other features such as the specific materials, support structures, surface treatment, or texture also could have influenced the participants’ evaluations, potentially minimizing the effect of the curvilinearity of the objects. Nevertheless, common-use objects tend to be heterogeneous in their features, and therefore some variability could be expected when using an ecologically valid stimuli set.

Finally, curvilinearity has been shown to facilitate the categorization of animate as opposite to inanimate objects (Long et al., 2017; Yetter et al., 2021). Animacy can be distinguished from shape cues, with a set of midlevel visual shape features, such as curvature and symmetry, playing an important role in the superordinate classification of objects along the animacy continuum (Schmidt et al., 2017). Therefore, future studies could explore how object animacy influences the curvature effect in preference judgement of biological (e.g., plants, animals, and humans), anthropomorphized (e.g., cars or objects with faces), or mechanically animated (e.g., robots) objects.

## Conclusions

In this study, we investigated the extent to which object contour (curvilinear vs. rectilinear) influences liking for commonly used objects within comparable categories (chairs vs. tables). Additionally, we examined whether participants’ visual inspection of object contours, measured through the number of fixations and mean fixation duration, varied by object category and how these patterns related to liking. Overall, our findings revealed no clear preference for curvature in either chairs or tables, with a limited effect of curvature on the estimated mean fixation duration. A plausible explanation for these results is that a high level of familiarization with specific classes of stimuli may lead to habituation and a subsequent decline in the preference for curvature (Biederman & Vessel, 2006; Tinio & Leder, 2009). Together, the results of this study align with the idea that curvature preference might be limited when considering more ecologically valid conditions.
